# Evaluating an Intervention to Increase Cereal Fiber Intake in Children: A Randomized Controlled Feasibility Trial

**DOI:** 10.1093/jn/nxaa347

**Published:** 2020-12-09

**Authors:** Angela S Donin, Claire M Nightingale, Michael R Perkin, Michael Ussher, Susan A Jebb, Rikard Landberg, Paul Welsh, Naveed Sattar, Peymane Adab, Chris G Owen, Alicja R Rudnicka, Derek G Cook, Peter H Whincup

**Affiliations:** Population Health Research Institute, St George's, University of London, London, United Kingdom; Population Health Research Institute, St George's, University of London, London, United Kingdom; Population Health Research Institute, St George's, University of London, London, United Kingdom; Population Health Research Institute, St George's, University of London, London, United Kingdom; Institute for Social Marketing and Health, University of Stirling, London, United Kingdom; Nuffield Department of Primary Care Health Sciences, Medical Sciences Division, University of Oxford, Oxford, United Kingdom; Division of Food and Nutrition Science, Chalmers University of Technology, Gothenburg, Sweden; Institute of Cardiovascular & Medical Sciences, University of Glasgow, Glasgow, United Kingdom; Institute of Cardiovascular & Medical Sciences, University of Glasgow, Glasgow, United Kingdom; Institute of Applied Health Research, University of Birmingham, Birmingham, United Kingdom; Population Health Research Institute, St George's, University of London, London, United Kingdom; Population Health Research Institute, St George's, University of London, London, United Kingdom; Population Health Research Institute, St George's, University of London, London, United Kingdom; Population Health Research Institute, St George's, University of London, London, United Kingdom

**Keywords:** Cereal fiber, children, type 2 diabetes risk, feasibility trial, dietary intervention

## Abstract

**Background:**

Observational studies have shown that higher cereal fiber intake is associated with reduced type 2 diabetes risk. However, it remains uncertain whether this association is causal.

**Objective:**

This study evaluated the feasibility of an intervention to increase cereal fiber intake in children using breakfast cereals.

**Methods:**

The study was a 2-arm parallel group randomized controlled trial in 9–10-y-old children, who received free supplies of high-fiber breakfast cereals (>3.5 g/portion) or low-fiber breakfast cereals (<1.0 g/portion) to eat daily for 1 mo with behavioral support to promote adherence. Children provided baseline and 1-mo fasting blood samples, physical measurements, and 24-h dietary recalls. The primary outcome was the group difference in change in plasma total alkylresorcinol (AR) concentration; secondary outcomes were group differences in nutrient intakes and adiposity indices. Analyses (complete case and multiple imputation) were conducted by regressing the final AR concentration on baseline AR in models adjusted for sex, ethnicity, age, and school (random effect).

**Results:**

Two-hundred seventy-two children were randomly assigned (137 receiving a low-fiber and 135 a high-fiber diet) and 193 (71%) provided fasting blood samples at baseline and follow-up. Among randomized participants, median (IQR) of baseline AR was 43.1 (24.6–85.5) nmol/L and of cereal fiber intake was 4.5 (2.7–6.4) g; 87% of participants reported consuming the cereal on most or all days. Compared with changes in the low-fiber group, the high-fiber group had greater increases in AR (40.7 nmol/L; 95% CI: 21.7, 59.8 nmol/L, *P* < 0.0001) and in reported cereal fiber intake (2.9g/d; 95% CI: 2.0, 3.7 g; *P* < 0.0001). There were no appreciable differences in other secondary outcomes.

**Conclusions:**

We have developed a simple and acceptable nutritional intervention that increases markers of daily cereal fiber intake in children. This intervention could be used to test whether increases in cereal fiber intake in children might reduce insulin resistance. This trial was registered at www.isrctn.com as ISRCTN33260236.

## Introduction

Foods high in dietary fiber have long been considered an important component of human diets and beneficial for good health, particularly gastrointestinal health. However, since the mid-20th century, observations that societies with very high-fiber diets had very low chronic disease risk (1) created interest in the possibility that dietary fiber may have wider health protection benefits. Over the past few decades, large prospective observational studies have reported that adults with high-fiber diets have lower risks of a range of chronic diseases, including cardiovascular disease, obesity, some cancers, and type 2 diabetes (2–6), in strongly graded associations (7). In the United Kingdom, in common with many other Western societies, intake of fiber is well below recommended levels (30 g fiber/d in adults, 20 g/d in children). National surveys have shown that adults need to increase their fiber intakes by around 50% for men and 75% for women to meet recommendations for good health; in children with lower intake recommendations, intakes would need to increase by more than one-third, with greater increases needed in children from lower socioeconomic groups (8). A population-wide intervention to increase dietary fiber intake could therefore have substantial public health benefits.

Recent systematic reviews and large-scale meta-analyses have suggested that for reducing risks of type 2 diabetes, higher intakes of cereal fiber may be particularly important compared with higher intakes of fiber from fruit or vegetables (7, 9, 10). However, compelling experimental evidence of a causal relationship between cereal fiber and type 2 diabetes is limited (11). Moreover, inconsistent definitions, doses, and durations have made reviewing the available evidence problematic (12). Trials that have focused specifically on increasing fiber intakes to reduce diabetes risk have tended to be small, with limited statistical power and often lacking objective data on adherence (13, 14). In contrast, large diabetes-prevention trials have often aimed to change multiple aspects of the diet and other health behaviors to achieve the greatest potential reduction in disease risk (15, 16), making it difficult to examine the causal role of individual dietary components. This limitation in the literature has been noted by several large-scale systematic reviews (7, 17). Well-designed, large-scale trials are urgently needed to robustly test the impact of cereal fiber on the risks of type 2 diabetes and its precursors (18). Before embarking on such a study, it is essential to develop interventions that are well tolerated and that lead to appreciable increases in cereal fiber intake.

We have previously shown that children who consume high-fiber breakfast cereals have lower insulin resistance (assessed using fasting insulin and the HOMA-IR) than children who ate a low-fiber breakfast (19), suggesting that the association between cereal fiber and emerging type 2 diabetes risk may be apparent in childhood. Improving diet quality in children offers the potential to establish healthy dietary habits that may persist into later life (20). However, there is currently little evidence in children on the effects of increasing cereal fiber intake on emerging type 2 diabetes risk. We therefore developed and evaluated a dietary intervention aiming to increase cereal fiber intake over a 1-mo period in 9–10-y-old children. Measuring dietary fiber intake is challenging; we therefore used a biomarker of whole-wheat fiber intake [fasting plasma total alkylresorcinol (AR)] to test whether the intervention had been successful in its primary aim to bring about a sustained change in dietary cereal fiber intake. Dietary recall data were collected to examine any further changes in dietary intakes, and biological markers were included to estimate secondary effects of dietary change (body composition and type 2 diabetes risk markers, e.g., insulin resistance). If shown to be effective, such an intervention could be used in future studies to examine the effects of increasing cereal fiber intake on insulin resistance and glycemic control.

## Subjects and Methods

### Study overview

The CRUNCH (CeReal nUtritioN for Child Health) study was a 2-armed parallel group randomized controlled feasibility trial investigating whether providing a free supply of high-fiber breakfast cereal (high-fiber group) to children currently consuming a low-fiber breakfast cereal would increase cereal fiber intake during a 1-mo period. As a feasibility trial for a larger full-scale trial, the 1-mo duration of the trial was chosen to represent a sustained dietary change of sufficient duration to bring about a change in metabolic outcomes such as fasting insulin and insulin resistance (21). The comparison group (low-fiber group) were also given a free supply of breakfast cereal but with a low fiber content. The study was based on use of commercially available high-fiber and low-fiber cereals, widely available from UK supermarkets (see **Supplemental Table 1** for details of cereals and their fiber contents). All participants received support and encouragement based on behavior change techniques (BCTs), detailed below. Ethical approval was granted by the St George's University Ethics Committee (SGREC17.0007). Written, informed parental consent was obtained separately for the initial assessments, which included a question on child allergies (detailed below), and for the feasibility trial; child assent was also obtained for all participants at entry to the study.

### Participants and initial procedures

A total of 23 London 0rimary schools agreed to take part in the study (70% response rate) between September 2017 and June 2018. All year 5 pupils in those schools (aged 9–10 y) were invited to take part in initial eligibility assessments, including a brief questionnaire on their current breakfast habits (including the name of their current breakfast cereal) and a taste test, which included all of the breakfast cereals (both low- and high-fiber) that would be offered in the feasibility trial. The study participants were also asked about parental and grandparental place of birth, used to define child ethnicity. Participants were asked to rate the palatability of each cereal they tasted on a 5-level Likert scale, with the following anchors; awful, not good, alright, good, and brilliant. Data from these initial assessments were used to identify children eligible for the trial. Inclusion criteria were as follows: currently eating a breakfast cereal with a low fiber content (<1-g cereal fiber per portion), scoring at least 1 of the high-fiber and 1 of the low-fiber cereals in the highest 2 Likert scale categories, and no relevant food allergies. This was to ensure that trial participants would be given breakfast cereals that they had previously tasted and found to be palatable. Children with diabetes were excluded from participation. Eligible children were given a briefing session about the trial by a member of the research team and were then given invitation letters and consent forms for the feasibility trial to take home to their parents/caregivers.

### Random assignment and interventions

After baseline assessment, eligible participants consenting to take part in the trial were individually randomly assigned to either receive a 1-mo free supply of high-fiber breakfast cereals (>3.5 g fiber per portion) or a 1-mo free supply of low-fiber breakfast cereals (<1 g of fiber per portion). Random assignment was carried out using the KCTU (King's Clinical Trials Unit) online randomization service, stratified by school and using a block size of 2 to maintain balanced random assignment to groups within schools. The study coordinator (not blinded) implemented the random assignment on the same day by preparing a 2-wk supply of the high- or low-fiber cereal preferred by each child in the taste test in plain packaging. This package was given out to each child at school ready to be taken home; children were not informed which cereal group they had been allocated to.

### Intervention Procedures

Participants in both the high-fiber and low-fiber groups were seen by the trial coordinator on the day of baseline assessments, when the trial coordinator provided a supply of the allocated cereal and implemented behavioral intervention procedures underpinned by the social cognitive theory of behavior change (22), which were coherent with standard BCT taxonomy (23) and designed to encourage daily breakfast cereal consumption (**Supplemental Table 2**).

The participants were given a “Participant Pack” at the end of the school day that included a 2-wk supply of their allocated cereal, a breakfast diary, a wall chart and stickers (to chart their progress), pens, pencils, fridge magnets, and badges. These items were used to provide reminders for the children to increase their adherence and engagement with the study. Participating children were asked to eat the cereal provided for them every day for 1 mo and to record in their breakfast diary what they ate for breakfast, including nonallocated items. Participants were given information on how to contact the research team, including via a study website (www.crunch.sgul.ac.uk) that had a message box they could use. The trial coordinator visited each school after 1 wk and administered a quiz about breakfast and general nutrition, to keep the participants interested in and engaged with the study. During this visit participants were asked whether the allocated cereal was still acceptable and the supply adequate; additional or replacement cereals were provided as needed. A further 2-wk supply of cereal was delivered to the children (via the primary school) halfway through the trial to take home.

### Outcome measures

Assessments were made at baseline and after 1 mo by research outcome assessors, who were blind to group allocation. On each occasion, each child was asked to provide a blood sample taken after an overnight fast and was asked about the last time of eating and drinking; s/he was then provided with breakfast. Measurements of height, weight, and body fatness (using a Tanita body composition analyzer BC-418 MA, Tanita Inc.) were made, followed by a 24-h recall dietary assessment using the Intake24 software program (www.intake24.co.uk). On the 1-mo visit, children were also given a short questionnaire. The questionnaire included a question asking how often the children had eaten the breakfast cereal, with the following 4 answer options: every day, most days, some days, and never/hardly ever; and a question asking if the children had enjoyed taking part in the experiment, with the following 5 answer options: I did not enjoy it at all, I did not really enjoy it, it was alright, I enjoyed taking part, I really enjoyed it.

The primary outcome was change in AR concentration (which is the sum of the C17:0, C19:0, C21:0, C23:0, and C25:0 homologues), a biomarker of whole-grain wheat and rye intakes, measured from fasting blood samples taken at baseline and at follow-up. Secondary outcomes included change in cereal fiber intakes measured through self-reported 24-h recalls and changes in dietary intakes of total energy; intakes of carbohydrates, fat, and protein; body weight; and body composition. Fasting measurements of plasma insulin, glucose, and blood lipids were made to inform the design of potential future outcome trials. Participants were defined as fasting if they reported consuming nothing other than water on the morning of assessment and had a plasma insulin concentration of >25 mU/L.

For laboratory analysis, blood samples were centrifuged at 1540 × *g* for 10 mins at room temperature and divided into aliquots within 6 h of collection; aliquots were then deep frozen at −70°C and transferred to central laboratories for measurement within 12 mo of collection. AR was measured using an LC-MS/MS method as described by Ross et al (24). Both baseline and follow-up samples from each child were analyzed in the same batch. Intra- and interbatch CVs were both <10%. Plasma total and HDL cholesterol and glucose were measured using an automated analyzer (c311, Roche Diagnostics); glycated hemoglobin was measured in whole blood using the same analyzer. Plasma insulin was measured with an automated immunoassay method (411, Roche Diagnostics), which does not cross-react with proinsulin.

### Study size and statistical analysis

We estimated that 100 participants with complete data in each group were required to have 90% power at the 1% significance level to detect a difference between the high-fiber and low-fiber groups of 0.7 SD in AR concentration at the end of the trial. We overrecruited by 35% to allow for withdrawals and for nonadherence with the requirement for fasting.

All analyses were carried out using STATA/SE software (Stata/SE 14 for Windows; StataCorp LP). Multilevel linear regression models were fitted using the mixed procedure to examine the effects of the high-fiber breakfast cereal intervention on concentrations of fasting AR and secondary outcomes in participants who received high-fiber breakfast cereal compared with controls who received low-fiber breakfast cereal, and the Wald test was used to examine statistical significance. Fasting AR at follow-up was regressed on fasting AR at baseline and in the intervention group, to estimate the change in AR between intervention groups efficiently while allowing for regression to the mean; school was adjusted for as a random effect in all analyses. The effect of further adjustment for participant characteristics, including sex, age (quartiles), and ethnic group (white European, black African origin, South Asian, and mixed/other; all fitted as fixed effects) was also examined. The same approach was used for secondary outcomes (cereal fiber intake; total energy intake; carbohydrate, protein, and fat intakes; body weight; fat mass; and fat mass percentage).

The primary analysis was a complete case analysis based on all children randomly assigned with data on the primary outcome (fasting AR) both at baseline and follow-up. A sensitivity analysis (effectively an intention to treat analysis) was carried out for the primary outcome by examining the impact of missing fasting AR data at baseline (*n* = 8) or follow-up (*n* = 59), using multiple imputation methods with chained equations (mi impute) using 40 simulations; 12 subjects with AR missing at both baseline and follow-up were excluded. Predictors included in the multiple imputation were AR at baseline or follow-up as well as sex, age quartiles, ethnic group, and cereal fiber intervention group, because these were potential predictors of missingness; 260 of 272 children randomly assigned were included in this imputation analysis. Finally, an exploratory per-protocol analysis was conducted that included only children who reported eating the cereal on most or all days.

## Results

**FIGURE 1:**
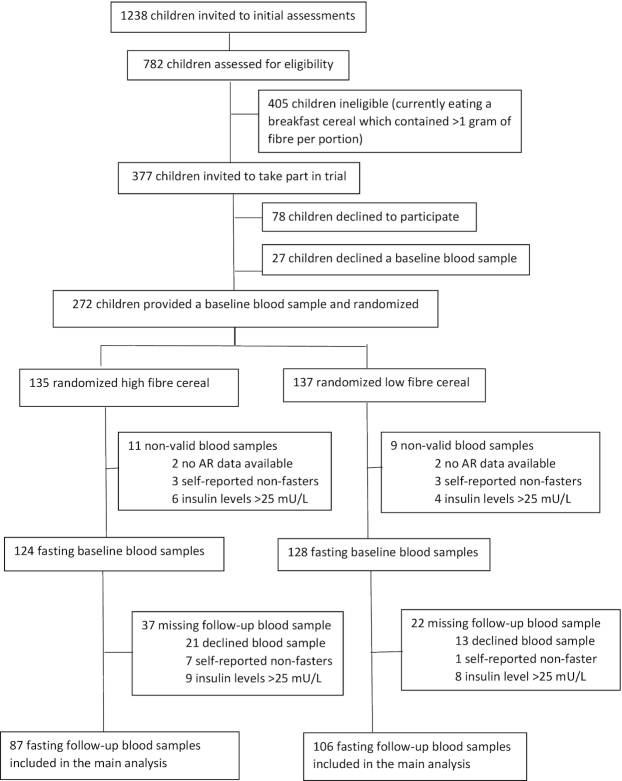
Trial participant recruitment, randomization, and follow-up. AR, plasma total alkylresorcinol.

**Figure 1** summarizes numbers of children recruited and randomly assigned into the study. Between September 2017 and June 2018, a total of 782 children took part in initial eligibility assessments (response rate of 63%). Of these 782 children, 377 (48%) children were eligible to take part in the trial (all reported that they were currently eating a low-fiber breakfast cereal and all scored ≥1 high-fiber and ≥1 low-fiber cereal in the top 2 palatability categories). In total, consent was received for 299 participants, of whom 272 children (72% of eligible) attended the baseline assessment and were randomly assigned (see Figure 1), 135 children were allocated to receive the high-fiber breakfast cereal, and 137 children were allocated the low-fiber breakfast cereal. Complete fasting AR data at baseline and 1 mo were available for 193 children who were included in the complete case analysis (87 in the high-fiber group and 106 in the low-fiber group); the sensitivity analysis for missing AR data was carried out in 260 children with at ≥1 AR value.

**Table 1** presents the baseline characteristics of the participants. There were more girls than boys; white European origin was the largest ethnic group, with smaller proportions of black Africans, South Asians, and other/mixed ethnicities. AR and reported cereal fiber intakes were slightly higher at baseline in the group allocated to high-fiber cereals, whereas protein and fat intakes were higher at baseline in the low-fiber group. Total energy intakes and the anthropometric measures did not differ markedly between the intervention groups.

**TABLE 1:** Baseline characteristics of all participants by intervention group^1^

	Intervention group		
	Low-fiber (*n* = 137)	High-fiber (*n* = 135)	All (*n* = 272)
Age, y	9.9 (9.6–10.3)	9.9 (9.6–10.2)	9.9 (9.6–10.2)
Sex, % female	56	62	59
Ethnicity, *n* (%)			
White European	60 (43.8)	53 (39.3)	113 (41.5)
Black African	19 (13.9)	26 (19.3)	45 (16.5)
South Asian	34 (24.8)	33 (24.4)	67 (24.6)
Other	24 (17.5)	23 (17.0)	47 (17.3)
Total energy intake,^2^ kcal/d	1322 (1,095–1,651)	1345 (1,067–1,676)	1344 (1,081–1,654)
Cereal fiber intake,^2^ g/d	4.2 (2.6–6.5)	4.7 (2.7–6.3)	4.5 (2.7–6.4)
Carbohydrate,^2^ g/d	194 (156–239)	196 (153–237)	194 (155–237)
Protein,^2^ g/d	53.3 (40.6–63.1)	50.5 (38.4–65.2)	51.1 (39.0–63.9)
Fat,^2^ g/d	47.0 (34.9–59.4)	43.2 (29.4–61.7)	44.8 (32.0–61.0)
Weight, kg	34.5 (29.8–41.5)	34.7 (30.7–40.9)	34.6 (30.3–41.1)
Fat mass,^3^ kg	7.7 (6.1–11.2)	8.1 (6.1–11.1)	7.9 (6.1–11.2)
Fat mass,^3^ %	23.0 (20.1–28.9)	23.1 (20.0–27.6)	23.0 (20.0–27.9)
Baseline fasting plasma analytes	*n* = 128	*n* = 124	*n* = 252
AR, nmol/L	42.1 (22.2–85.7)	43.8 (26.4–82.5)	43.1 (24.6–85.5)
Insulin, mU/L	6.8 (4.6–10.3)	7.1 (4.9–9.0)	7.0 (4.8–10.0)
Glucose,^4^ mmol/L	4.5 (4.2–4.7)	4.5 (4.3–4.7)	4.5 (4.2–4.7)
LDL cholesterol, mmol/L	2.0 (1.7–2.4)	2.0 (1.5–2.5)	2.0 (1.6–2.4)
HDL cholesterol, mmol/L	1.4 (1.2–1.7)	1.4 (1.2–1.7)	1.4 (1.2–1.7)
TG, mmol/L	0.6 (0.5–0.8)	0.6 (0.5–0.8)	0.6 (0.5–0.8)
Vitamin C,^5^ μmol/L	67.4 (52.6–83.0)	69.4 (56.2–81.0)	68.4 (54.6–81.4)
Baseline fasting HbA1c,^6^ mmol/mol	33.1 (31.4–35.1)	33.2 (31.7–34.7)	33.1 (31.6–34.7)

1 Values represented are median (IQR) or frequency (%) unless otherwise indicated. AR, plasma total alkylresorcinol; HbA1c, glycated hemoglobin; TG, triglyceride.

2 Missing data: low-fiber: *n* = 6, high-fiber: *n* = 1.

3 Missing data: high-fiber: *n* = 1.

4 Missing data: low-fiber: *n* = 3, high-fiber: *n* = 3.

5 Missing data: low-fiber: *n* = 6, high-fiber: *n* = 6.

6 Missing data: low-fiber: *n* = 3, high-fiber: *n* = 1.

Most children (87%) reported eating the allocated cereal on most or all days through the intervention period, although this proportion was higher in the low-fiber group (94%) than the high-fiber group (80%) (mean difference: 13.8%; 95% CI: 9.8, 18.7%). **Table 2** presents differences in changes in the primary and secondary outcomes during the 1-mo trial period between the high-fiber group and the low-fiber group in the complete case analysis. All analyses included a term for school (random effect) and model 2 also included adjustments for sex, age quartiles, and ethnic group (fixed effects). At follow-up, the high-fiber cereal intervention group was associated with a significantly greater adjusted increase in fasting AR compared with changes in the low-fiber cereal group and a greater adjusted increase in self-reported cereal fiber intakes. There were no changes in any of the other secondary outcomes measured, including dietary intakes (total energy, carbohydrate, fat, and protein) and indices of adiposity [body mass index (BMI): kg/m^2^; or fat mass index]. Multiple imputation was used to impute fasting AR values that were missing either at baseline (*n* = 8) or at follow-up (*n* = 59) and included in the analysis; 12 children with missing AR data at both time points were excluded from this analysis. In this analysis, increases in fasting AR observed in the high-fiber cereal group were very similar to those in the low-fiber group that were observed in the complete case analysis. Differences in changes in the blood-based risk markers over the study period between the 2 groups were examined (**Table 3**); no significant differences were observed.

**TABLE 2:** Effect of a high-fiber cereal intervention compared with low-fiber cereal on plasma AR, dietary intake, weight, and adiposity in participants who provided baseline and follow-up data: complete case analysis^1^

	Effect of high-fiber intervention	
	Mean difference (95% CI)	*P* value
AR, nmol/L (*n* = 193)		
Model 1	41.6 (21.7, 61.5)	<0.0001
Model 2	40.7 (21.7, 59.8)	<0.0001
Cereal fiber, g/d (*n* = 252)		
Model 1	2.9 (2.0, 3.8)	<0.0001
Model 2	2.9 (2.0, 3.7)	<0.0001
Energy intake, kcals/d (*n* = 252)		
Model 1	44.6 (−80.6, 169.8)	0.49
Model 2	47.3 (−79.6, 174.1)	0.47
Carbohydrate, g/d (*n* = 252)		
Model 1	2.0 (−16.3, 20.3)	0.83
Model 2	1.6 (−16.9, 20.1)	0.86
Protein, g/d (*n* = 252)		
Model 1	−0.1 (−5.6, 5.3)	0.96
Model 2	0.1 (−5.5, 5.7)	0.97
Fat, g/d (*n* = 252)		
Model 1	4.0 (−2.4, 10.4)	0.22
Model 2	4.4 (−2.0, 10.8)	0.18
Weight, kg (*n* = 261)		
Model 1	0.0 (−0.1, 0.2)	0.67
Model 2	0.0 (−0.1, 0.2)	0.63
Fat mass, kg (*n* = 261)		
Model 1	0.0 (−0.2, 0.2)	0.87
Model 2	0.0 (−0.2, 0.2)	0.88
Fat mass, % (*n* = 261)		
Model 1	0.0 (−0.3, 0.4)	0.87
Model 2	0.0 (−0.4, 0.4)	0.92

1 Model 1, outcome at follow-up was regressed on outcome at baseline and intervention group with adjustment for school (random effect); model 2, outcome at follow-up was regressed on outcome at baseline and intervention group with adjustment for school (random effect), age (quartiles), sex, ethnic group (fixed effects). *P* values are based on a Wald test for statistical significance. AR, plasma total alkylresorcinol.

**TABLE 3:** Effect of high-fiber cereal intervention compared with low-fiber cereal on blood markers in participants who provided baseline and follow-up data: complete case analysis^1^

	Effect of high-fiber intervention	
	Difference (95% CI)	*P* value
Plasma insulin, mU/L (*n =* 193)		
Model 1	−0.1 (−1.0, 0.8)	0.85
Model 2	−0.1 (−1.0, 0.8)	0.81
Whole blood HbA1c, mmol/mol (*n =* 189)		
Model 1	−0.3 (−0.8, 0.3)	0.37
Model 2	−0.3 (−0.9, 0.3)	0.32
Plasma glucose, mmol/L (*n =* 188)		
Model 1	0.0 (−0.1, 0.1)	0.84
Model 2	0.0 (−0.1, 0.1)	0.74
Plasma LDL cholesterol, mmol/L (*n =* 193)		
Model 1	−0.1 (−0.2, 0.0)	0.18
Model 2	−0.1 (−0.1, 0.0)	0.27
Plasma HDL cholesterol, mmol/L		
Model 1 (*n =* 193)	0.0 (−0.1, 0.1)	0.74
Model 2	0.0 (0.0, 0.1)	0.53
Plasma TG, mmol/L (*n =* 193)		
Model 1	0.0 (0.0, 0.1)	0.47
Model 2	0.0 (0.0, 0.1)	0.33
Plasma vitamin C, μmol/L (*n =* 186)		
Model 1	0.4 (−4.4, 5.2)	0.87
Model 2	0.8 (−3.9, 5.6)	0.73

^1^Model 1, outcome at follow-up was regressed on outcome at baseline and intervention group with adjustment for school (random effect); model 2, outcome at follow-up was regressed on outcome at baseline and intervention group with adjustment for school (random effect), age (quartiles), sex, ethnic group (fixed effects). *P* values are based on a Wald test for statistical significance. AR, plasma total alkylresorcinol; HbA1c, glycated hemoglobin; TG, triglyceride.

At follow-up, most children (60%) reported that they had “really enjoyed taking part,” and 25% reported that they had “enjoyed taking part,” with the remaining children reporting that “it was alright” (12%) or “did not really enjoy it” (3%). Similar proportions of acceptability were recorded for both high-fiber and low-fiber groups.

## Discussion

An intervention that provided free supplies of palatable high-fiber breakfast cereals to 9–10-y-old children and free supplies of palatable low-fiber cereals for the control group, with support and encouragement provided to both groups to eat breakfast based on recognized BCTs, successfully increased cereal fiber intakes in the intervention group over a 1-mo period. This result was documented both by a difference in change in AR (appreciably higher in the high-fiber group than in the low-fiber group following the 1-mo trial period) and by data from a 24-h recall taken at the end of the 1-mo period, showing that nearly 3 g more cereal fiber was consumed in the high-fiber group than the low-fiber group, with no other changes observed in dietary intakes or adiposity measures between the 2 groups. Adherence (children who recalled eating the breakfast cereal every day or most days) was good, although slightly lower in the high-fiber group than in the low-fiber group.

This intervention in children was unique in its design; the main aim was to change cereal fiber intake without altering other components of the diet, through the provision of replacement foods rather than nutritional supplements. This result appears to have been successfully achieved, with changes in cereal fiber in the high-fiber group (documented both by changes in AR and dietary recall) achieved without changes in energy intake, macronutrient or micronutrient intake, or weight and other anthropometric measures. Previous intervention trials in children have tended to be multicomponent and have changed numerous aspects of the diet, including fiber intakes, to achieve an improvement in health outcomes (25–27). Often these approaches led to changes in total energy intakes and weight status (26). Some previous interventions that aimed to increase fiber intakes were unsuccessful (26, 27), even when substitute foods were provided (28), results emphasizing the importance of the behavioral support to encourage consumption of the breakfast cereal. The BCTs used were based on the key constructs of the social cognitive theory of behavior change (22). The aim of using the BCTs was to increase self-efficacy and self-regulation and create environmental triggers to help to remind and motivate the participants to eat the breakfast cereal daily throughout the study period. The BCTs were applied in both groups to maximize the achieved difference in cereal fiber intake we might achieve; this also meant that both researchers and participants could remain blind to the allocated cereal groups. Although the participants may have been able to identify the cereal they were eating (a common issue for dietary interventions), they were unaware of the trial aim to increase fiber intakes in the high-fiber group.

### Strengths and limitations

A particular strength of this trial was the use of an objective marker of whole-wheat cereal fiber intakes (AR) in conjunction with subjective self-reported measures to compare the changes in intakes in the different breakfast cereal groups; both measures consistently showed higher cereal fiber intakes in the high-fiber intervention group. Reassuringly, these differences in the primary outcomes suggest that any contamination that took place between the intervention and control groups was limited (in the light of the marked differences in cereal fiber intake observed). Participants were individually randomly assigned (rather than using a cluster randomly assigned design) for greater statistical efficiency. The consistency of the AR differences between the high- and low-fiber groups in the complete case analysis and the imputation (intention to treat) analysis is reassuring. However, we acknowledge that even the missing at random analyses cannot guard against data that are not missing at random, conditional on the covariates. ARs are phenolic lipids that are present in the bran fraction of wheat and rye, and once consumed they are absorbed and detectable in plasma. ARs have therefore been suggested as potential biomarkers of whole-grain wheat and rye intakes (29). There are clear advantages of using objective markers of dietary intakes and, although not a precise measure of cereal fiber intake at an individual level, AR provides both a degree of ranking in individuals (28, 29) and an objective marker of recent cereal fiber intake from whole wheat and rye at a group level (30). In the current trial, participants were asked to consume whole-wheat breakfast cereals, therefore consuming all parts of the grain, and the measure of AR was used solely as a measure of adherence to the intervention and not to examine any potential relation between AR and metabolic outcomes. In relation to previous studies, it is difficult to compare AR concentrations across different age groups, as absorption, distribution, and/or elimination appear to be highly influenced by age (higher AR values in younger children). Plasma AR concentrations in the present study were of a similar magnitude, though slightly lower than those in 8–10-y-old Danish children with higher whole-grain intakes (31).

This feasibility study was not powered to detect moderate intervention effects on insulin resistance, glycemic control, or other markers of cardiometabolic risk, which showed no statistically significant differences. However, potentially meaningful favorable population-wide changes in these markers (for example, a reduction in mean fasting insulin of 1.0 mU/L) could not be confidently excluded. Thus, a substantially larger trial (estimated sample size of ∼1400 participants based on data from this study) would be needed to investigate the efficacy of this intervention on fasting markers of insulin resistance that were of a magnitude consistent with those in our earlier observational study (19). Although the current investigation suggested that large numbers of participants would need to be screened to identify eligible children, >70% of participants completed the trial, a rate which could potentially be increased with a strong focus on the importance of fasting and successful blood sampling. It is also possible that the use of much larger doses of fiber (through supplements) and the use of maximally stimulated measures of insulin resistance (assessed with euglycemic hyperinsulinemic clamps) could reduce the size of the trial needed, though these changes would increase the trial's invasiveness and would probably limit its practical feasibility.

In summary, we have developed an acceptable intervention that appreciably increases markers of daily cereal fiber intakes in children. Given the current low population intakes of cereal fiber in this age group, an intervention that increases cereal fiber by up to 3 g/d could have widespread public health relevance and applicability. Future research is needed to see if this change in diet leads to an improvement in markers of type 2 diabetes and can be sustained in the longer term.

## Supplementary Material

nxaa347_Supplemental_File

## Notes

Funded by the Wellcome Trust Seed Award in Science (200611/Z/16/Z). CMN is supported by the Wellcome Trust Institutional Strategic Support Fund (204809/Z/16/ Z) awarded to St George's, University of London. SAJ is a National Institute for Health Research (NIHR) senior investigator and is funded by the NIHR Oxford Biomedical Research Centre (BRC) Obesity, Diet and Lifestyle Theme and NIHR Applied Research Collaboration (ARC) Oxford and Thames Valley. This study was supported by the National Institute for Health Research (NIHR) Applied Research Collaboration South London (NIHR ARC South London). The views expressed are those of the author(s) and not necessarily those of the NIHR or the Department of Health and Social Care.

Author disclosures: The authors report no conflicts of interest.

Data described in the manuscript, code book, and analytic code will be made available upon request pending application and approval.

Supplemental Tables 1–3 are available from the “Supplementary Data” link in the online posting of the article and from the same link in the online table of contents at https://academic.oup.com/jn.

Abbreviations used: AR, plasma total alkylresorcinol; BCT, behavior change technique; HOMA-IR, homeostasis model assessment of insulin resistance.
