# Supplementary material for: Evaluating an Intervention to Increase Cereal Fiber Intake in Children: A Randomized Controlled Feasibility Trial
Source: J Nutr. 2020 Dec 9;151(2):379–86. doi: 10.1093/jn/nxaa347 (PMC7849987; doi:10.1093/jn/nxaa347)
Supplement: nxaa347_Supplemental_File [file nxaa347_supplemental_file.docx]

**Evaluating an intervention to increase cereal fiber intake in children: a randomized controlled feasibility trial. (**AS Donin)

On-line Supplementary Material

Supplementary Table 1: Breakfast cereals included in the trial

|  | Breakfast cereal (suggested serving) | Fiber content per serving | Energy content per serving | Fiber content per 100g | Energy content per 100g |
| --- | --- | --- | --- | --- | --- |
|  |  |  |  |  |  |
| High Fiber Cereals: | Weetabix (2 biscuits) | 3.8g | 136kcal | 10g | 362kcal |
|  | Wholegrain apricot wheats (per 45g) | 3.8g | 151kcal | 8.3g | 335kcal |
|  | Malted wheats (per 40g) | 4.3g | 144kcal | 10.9g | 360kcal |
|  | Crunchy bran (per 40g) | 8.0g | 140kcal | 22g | 351kcal |
| Low Fiber Cereals: | Rice Crispies (per 30g) | 0.6g | 116kcal | 2.0g | 387kcal |
|  | Cornflakes (per 30g) | 0.9g | 113kcal | 3.0g | 378kcal |
|  | Balance (per 30g) | 0.8g | 114kcal | 2.7g | 382kcal |

Supplementary Table 2: Key constructs, behaviour change techniques and study activities they relate to.

| **Key constructs** | **Behaviour change technique definition (23)** | **Activity** |
| --- | --- | --- |
| **Self-Efficacy**  Confidence in the ability to consume the designated breakfast cereal on a daily basis. | Action Planning | -Introduce breakfast cereals using taster sessions to assess palatability and preferences  -Tailor the participant’s individual choice of cereal for the duration of the trial to ensure the intervention is enjoyable for the participant and increase motivation to succeed  -Provide free breakfast cereals  -Recommendation to eat allocated cereal every day for one month |
| **Social Support**  Extent to which support from family members and research team aids in behaviour change | Plan social support/social change | -Identify and encourage appropriate social support (e.g., positive reinforcement from family, family monitoring of progress on wall chart)  -school visit from researcher mid-trial |
| **Self-regulation**  Learning skills which increase motivation towards the desired breakfast behaviour and increase ability to resist alternative behaviour, such as alternative cereals or not eating breakfast | Barrier identification/problem solving  Teach to use prompts/cues  Prompt self-monitoring of behaviour | -Evaluation and reflection (information and education given by research team at baseline, mid-point and on completion; participation pack to encourage and motivate continued behaviour change)  -Self monitoring (breakfast diary, wall chart with stickers) |
| **Outcome expectations**  Having a strong belief in the value of switching to the designated cereal | Provide information on consequences of behaviour in general | -Provide information/education  -Message: “Eating breakfast every day is important for health. You are helping us with important research to investigate the effect of the content of breakfast cereal on your health” |
| **Environmental factors**  Create an environment which fosters behaviour change | Environmental restructuring  Use of follow-up prompts | -Create triggers in environment (wall chart; fridge magnets, cereal out and ready the night before; study bowl; school visit from researcher mid-trial) |
| **Motivation**  Maintain a strong desire to continue behaviour change | Stimulate anticipation of future rewards | -Put in place personalised rewards  (offer of gift vouchers on completion and return of wall chart and diary) |

Supplementary table 3: Baseline characteristics by intervention group for 193 subjects with fasting measures of plasma AR at baseline and follow-up^1^

|  | Intervention group, median (IQR) | | | | | |
| --- | --- | --- | --- | --- | --- | --- |
|  | Low fiber | | High fiber | | All | |
|  | n=106 | | n=87 | | n=193 | |
| Age, y | 9.9 | (9.6, 10.2) | 9.9 | (9.6, 10.2) | 9.9 | (9.6, 10.2) |
| Sex, % female | 57% | | 62% | | 59% | |
| Ethnicity, n (%) |  |  |  |  |  |  |
| White European | 50 | (47.2%) | 39 | (44.8%) | 89 | (46.1%) |
| Black African | 9 | (8.5%) | 15 | (17.2%) | 24 | (12.4%) |
| South Asian | 27 | (25.5%) | 21 | (24.1%) | 48 | (24.9%) |
| Other | 20 | (18.9%) | 12 | (13.8%) | 32 | (16.6%) |
| Total energy intake^2^, kcal/d | 1,376 | (1,095, 1,668) | 1,279 | (1,050, 1,685) | 1,326 | (1,069, 1,670) |
| Cereal Fiber intake^2^, g/d | 4.2 | (2.6, 6.6) | 4.6 | (2.9, 6.6) | 4.5 | (2.8, 6.6) |
| Carbohydrate^2^, g/d | 195 | (156, 236) | 188 | (149, 238) | 193 | (150, 237) |
| Protein^2^, g/d | 53.4 | (40.6, 63.1) | 49.3 | (36.8, 66.5) | 50.7 | (38.7, 64.7) |
| Fat^2^, g/d | 45.0 | (35.8, 61.0) | 43.1 | (28.5, 64.9) | 44.1 | (32.3, 61.7) |
| Weight, kg | 33.3 | (29.4, 40.5) | 34.3 | (30.7, 39.1) | 33.8 | (30.2, 39.7) |
| Fat mass, kg | 7.5 | (5.8, 10.8) | 7.9 | (6.1, 9.9) | 7.6 | (6.1, 10.1) |
| Fat mass, % | 22.9 | (19.8, 27.6) | 22.5 | (20.0, 26.7) | 22.7 | (19.9, 26.7) |
| Baseline fasting plasma analytes |  |  |  |  |  |  |
| Total AR, nmol/L | 42.7 | (21.9, 86.8) | 44.4 | (26.6, 89.5) | 43.2 | (24.3, 87.3) |
| Insulin, mU/L | 6.5 | (4.5, 10.2) | 7.1 | (5.0, 8.8) | 6.8 | (4.8, 9.5) |
| Glucose^3^, mmol/L | 4.5 | (4.2, 4.7) | 4.5 | (4.3, 4.7) | 4.5 | (4.2, 4.7) |
| LDL cholesterol, mmol/L | 2.0 | (1.7, 2.4) | 2.0 | (1.5, 2.5) | 2.0 | (1.6, 2.4) |
| HDL cholesterol, mmol/L | 1.5 | (1.2, 1.7) | 1.4 | (1.2, 1.7) | 1.4 | (1.2, 1.7) |
| TGs, mmol/L | 0.6 | (0.5, 0.7) | 0.6 | (0.5, 0.8) | 0.6 | (0.5, 0.8) |
| Vitamin C^4^, µmol/L | 67.8 | (52.6, 84.0) | 69.4 | (59.6, 81.0) | 69.4 | (55.8, 82.6) |
| Baseline fasting HbA1c^5^, mmol/mol | 33.1 | (31.4, 35.1) | 33.2 | (31.8, 34.7) | 33.1 | (31.6, 34.7) |

^1^Values represented are median (IQR) or frequency (%)

Missing data: ^2^ low fiber: n=4; ^3^ low fiber: n=1, high fiber: n=1; ^4^ low fiber: n=3, high fiber: n=2; ^5^ low fiber: n=1;
